# Neutral Processes Provide an Insight Into the Structure and Function of Gut Microbiota in the Cotton Bollworm

**DOI:** 10.3389/fmicb.2022.849637

**Published:** 2022-05-03

**Authors:** Sali Li, Rui Tang, Hao Yi, Zhichao Cao, Shaolei Sun, Tong-Xian Liu, Sicong Zhang, Xiangfeng Jing

**Affiliations:** ^1^State Key Laboratory of Crop Stress Biology for Arid Areas, College of Plant Protection, Northwest A&F University, Xianyang, China; ^2^Key Laboratory of Integrated Pest Management on the Loess Plateau of Ministry of Agriculture, College of Plant Protection, Northwest A&F University, Xianyang, China; ^3^Shandong Academy of Pesticide Sciences, Jinan, China

**Keywords:** neutral model, microbiota, community, diversity, *Helicoverpa armigera*

## Abstract

Gut-associated microbes can influence insect health and fitness. Understanding the structure of bacterial communities provides valuable insights on how different species may be selected and their functional characteristics in their hosts. The neutral model is powerful in predicting the structure of microbial communities, but its application in insects remains rare. Here, we examined the contribution of neutral processes to the gut-associated bacterial communities in *Helicoverpa armigera* caterpillars collected from different maize varieties at four locations. The gut-associated bacteria can be assigned to 37 Phyla, 119 orders, and 515 genera, with each individual gut containing 17–75% of the OTUs and 19–79% of the genera in the pooled samples of each population. The distribution patterns of most (75.59–83.74%) bacterial taxa were in good agreement with the neutral expectations. Of the remaining OTUs, some were detected in more individual hosts than would be predicted by the neutral model (i.e., above-partition), and others were detected in fewer individual hosts than predicted by the neutral model (i.e., below-partition). The bacterial taxa in the above-partitions were potentially selected by the caterpillar hosts, while the bacteria in the below-partitions may be preferentially eliminated by the hosts. Moreover, the gut-associated microbiota seemed to vary between maize varieties and locations, so ecological parameters outside hosts can affect the bacterial communities. Therefore, the structure of gut microbiota in the *H. armigera* caterpillar was mainly determined by stochastic processes, and the bacteria in the above-partition warrant further investigation for their potential roles in the caterpillar host.

## Introduction

Lepidoptera is the second largest insect order, and many of them are the most devastating pests of the agricultural and forest ecosystems worldwide (Sree and Varma, 2015). Diverse microbial communities inhabiting caterpillar guts have important impacts on host biology (Engel and Moran, 2013; Paniagua Voirol et al., 2018), such as food digestion and nutrient acquisition (Pinto-Tomás et al., 2007; Anand et al., 2010; Xia et al., 2017), pesticide resistance (Xia et al., 2018; Chen et al., 2020), and host interactions with pathogens (Takatsuka and Kunimi, 2000; Broderick et al., 2006; Shao et al., 2017). Gut-associated microbiota are also actively involved in the interactions between caterpillars and their host plants. For example, the gut flora in caterpillars can alter plant response by secreting bioactive molecules to directly act upon plants (Spiteller et al., 2000; Indiragandhi et al., 2008), or regulating the secretion of caterpillar salivary elicitor to induce plant defense (Wang et al., 2017). On the other hand, there are many parasitic or pathogenic microbes in caterpillar gut, and *Bacillus thuringiensis* is probably the best-known species (Broderick et al., 2006; Mukherjee et al., 2010; Shao et al., 2017; Chevrette et al., 2019).

In contrast to the low-diversity bacterial communities in the gut of sap-feeding insects, species richness and relative abundance of gut associated bacteria are much higher in caterpillars (Jing et al., 2014; Santos-Garcia et al., 2020). So far, most research tended to characterize the most common microbial taxa associated with caterpillars, and to evaluate the fitness contribution of the most abundant bacteria to caterpillar hosts (Broderick et al., 2004; Xiang et al., 2006; Robinson et al., 2010; Pinto-Tomás et al., 2011; Xia et al., 2013; Chen et al., 2018; Jones et al., 2019). However, host fitness contributed by the associated microbiota is usually the outcome of the whole bacterial community (Gould et al., 2018). Therefore, it is essential to understand the processes and dynamics of gut microbiota assembly among individual hosts.

The neutral theory of biodiversity is a mechanistic model for predicting species coexistence and biodiversity patterns in ecological communities. It was initially developed to predict ecological communities of large organisms such as plants and animals, and recently applied to microbial community ecology (Bell et al., 2005; Sloan et al., 2006; Woodcock et al., 2007; Caruso et al., 2011; Levy and Borenstein, 2013; Morris et al., 2013; Nemergut et al., 2013; O’Dwyer et al., 2015; Venkataraman et al., 2015; Li and Ma, 2016). In contrast to the traditional niche theory (deterministic and selective processes), which predicts that species with different properties occupy niches with different characters, the neutral theory assumes that stochastic processes, independent of host traits, play important roles in shaping microbial community composition in an individual host. In other words, it assumes microbial species in a community are functionally equivalent, and the assemblages are driven by random dispersal and birth/death events (Costello et al., 2012). Therefore, taxa that are abundant in the intestinal communities of all individuals are more likely to disperse by chance and be randomly sampled by an individual host, and less abundant taxa can be lost from an individual host due to ecological drift. The microbes deviating from the neutral model can be good candidates worthy of further investigation for their potential roles in a host (Sieber et al., 2019). The structure of *Drosophila* gut microbiota has been extensively investigated since the early work by Wong et al. (2011), and the recent application of the neutral model further provided key insights into the processes shaping bacterial communities in these model organisms (Martinson et al., 2017; Adair et al., 2018). However, evaluation of the contribution of the neutral processes to insect microbiota community structures remains rare.

In contrast to previous research based on the pooled samples (Mishra and Tandon, 2003; Xiang et al., 2006; Priya et al., 2012; Tang et al., 2012; Ranjith et al., 2016; Shinde et al., 2017), we profiled gut-associated bacteria in individual *H. armigera* larvae collected from maize plants at four locations to address two aims in this study. First, the contribution of neutral processes to the gut microbiota community associated with the individual caterpillar was evaluated. As far as we know, no investigation of neutral processes has been conducted for microbial communities in caterpillars. Second, we examined biodiversity patterns in microbial communities and ecological determinants of variation among individual caterpillar hosts.

## Materials and Methods

### Sample Preparation

**Figure 1:**
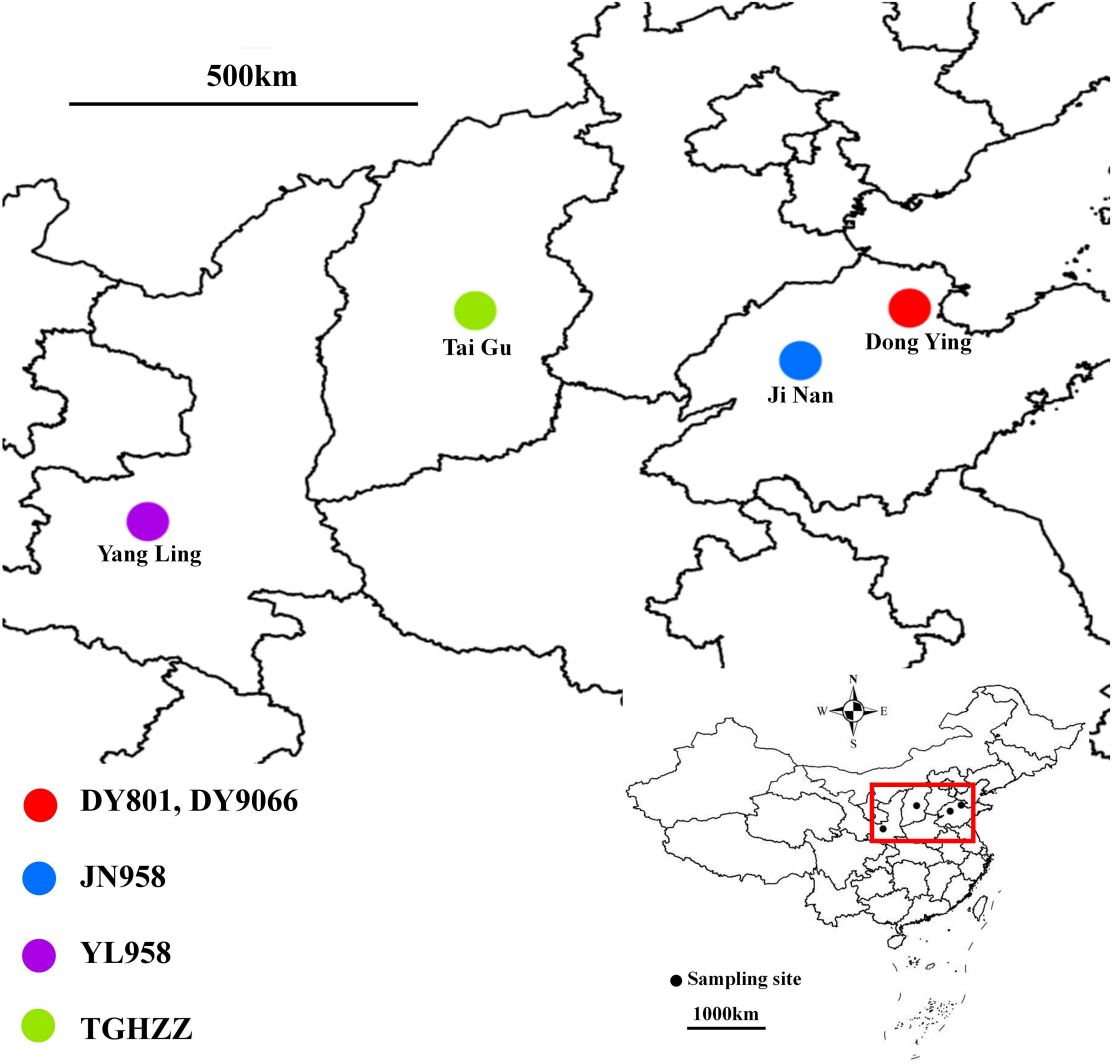
Collection sites of the five caterpillar populations in this study. DY801 and DY9066 referred to the two populations being collected on two maize varieties (Ludan 801 and Ludan 9,066) at one site, Dong Ying, Shandong. JN958 and YL958 referred to the two populations being collected on a single maize variety (Zhengdan 958) at two sites, Ji Nan, Shandong and Yang Ling, Shaanxi. TGHZZ referred to the population being collected on the maize variety of Heizhenzhu in the city of Tai Gu in Shanxi province.

*Helicoverpa armigera* larvae were collected from four maize varieties at four locations across three provinces of China in September 2018 (Supplementary Table S1; Figure 1). At one location (Dong Ying, Shandong), caterpillars were collected from two varieties, Ludan 801 and Ludan 9,066, planted at the adjacent sites. These two populations were labelled as DY801 and DY9066. We also collected caterpillars from a single variety (Zhengdan 958) at two locations (Ji Nan, Shandong and Yang Ling, Shaanxi), and we labelled them as JN958 and YL958. Another population (TGHZZ) was collected from a different maize variety (Heizhenzhu) in Tai Gu, Shanxi. The larvae were collected in corn ears and individually put in a sterile plastic tube containing corn ear silk from the collecting corn. All larvae were delivered to laboratory right away. Once arrived, the insects were prepared for dissection. First, all insects were starved for 4 h to discharge the food in the gut (Brinkmann and Tebbe, 2007; Adair and Douglas, 2017). Second, each insect was individually rinsed with 5‰ NaClO, 70% ethanol and sterile water. Third, each caterpillar was placed on an ice-cold clean glass slide with a clean tweezer, and its head was cut off with a clean scissor. Fourth, the gut was dissected out from the body and transferred into a 1.5 ml RNase/Dnase-free tube. Fifth, the tube containing the gut was immediately placed in liquid nitrogen. All the operations were conducted under a laminar flow hood. Tubes containing the gut were stored at −80°C until DNA extraction.

### Bacterial DNA Extraction and PCR Amplification

Genomic DNA was extracted from each gut using cetyltrimethylammonium bromide (CTAB) DNA extraction method (Doyle and Doyle, 1990), and used for PCR amplification. Briefly, each gut was homogenized in 1 ml sterile CTAB buffer containing 20 mg lysozyme, and incubated at 65°C for 1 h. DNA was extracted with equal volumes of phenol (pH 8.0): chloroform: isoamyl alcohol (25:24:1) and chloroform: isoamyl alcohol (24:1). Each sample was mixed thoroughly and centrifuged at 12000 rpm for 10 min at 4°C, and the aqueous phase was transferred to a new tube. Isopropanol was added to precipitate DNA and all samples were left at −20°C overnight. Each sample was then centrifuged at 12000 rpm for 10 min. The precipitate was washed twice with 1 ml of 75% ethanol, air-dried, and resuspended in 50 μl sterile endonuclease-free water. DNA extraction was incubated with RNase A at 37°C for 15 min to remove RNA contamination. DNA concentration was determined with a NanoDrop^™^ 2000 (Thermo Fisher Scientific, San Jose, CA, USA) and then diluted to 10 ng/μl for sequencing. The PCR primers, 515F/806R, were used to amplify the V4 hypervariable region of 16S rRNA gene (Donkersley et al., 2018; Wang et al., 2018). The PCR reaction mixtures (30 μl) contained 15 μl of Phusion^®^ High-Fidelity PCR Master Mix (New England Biolabs), 0.2 uM of forward and reverse primers, and about 10 ng template DNA. Thermal cycling consisted of initial denaturation at 98°C for 1 min followed by 30 cycles of 98°C for 10 s, 55°C for 30 s and 72°C for 30 s, with 5 min final extension at 72°C. Products were detected and excised from 2% agarose gel and purified with GeneJET^™^ Gel Extraction Kit (Thermo Scientific). Sterile water was included as a negative control for extraction and amplification contamination.

### Bacterial DNA Sequencing

Sequencing libraries were generated using Ion Plus Fragment Library Kit 48 rxns (Thermo Scientific), and the library quantity was assessed on the Qubit^®^ 2.0 Fluorometer (Thermo Scientific). Multiplexed, single-end sequencing was performed on an Ion S5TM XL platform (600 bp) in Tianjin Novogene Bioinformatic Technology Co., Ltd., China. Sequence data were uploaded to NCBI Sequence Read Archive under accession numbers BioProject PRJNA769459.

Low-quality reads were screened out according to the Cutadapt (V1.9.1)^1^ quality-controlling process, and chimera sequences were deleted by comparing with the reference database (Silva database)^2^ through UCHIME algorithm.^3^ Single-end reads with 253 bp average length were assigned to samples based on their unique barcode, and the barcode and primer sequence were then removed. The clean reads with the similarity of ≥97% were assigned to the same OTUs (operational taxonomic units). Representative sequence for each OTU was annotated against Silva Database (see footnote 2) based on Mothur algorithm. Chloroplast sequence was removed, and the OTUs with <100 reads were excluded.

### Statistical Analysis

OTUs abundance was normalized by rarefying (random subsampling without replacement) from each sample to the number of reads in the sample with the least number of sequences. Subsequent analyses were all performed based on the normalized data. A Venn diagram was made by an online tool^5^ to identify the unique and shared OTUs in the gut of all *Helicoverpa armigera* caterpillars from the five populations. Bacterial diversity estimates, including OTU richness, Shannon and Simpson, were calculated with QIIME (Version1.7.0) and displayed with R software (Version 2.15.3; R Core Team, 2021).^6^ Statistical significance of variation in diversity measures between different populations was assessed by the Wilcox rank sum test. Principal coordinate analysis (PCoA) was used to evaluate microbial community structure based on weighted Unifrac distances. Then, we used R “vegan” package to conduct Anosim and Adonis analysis based on Bray–Curtis distance. Taxa with significant differences between different populations were compared by linear discriminatory analysis effective size (LEfSe) analysis.

The Sloan neutral community model was used to assess the importance of stochastic processes in the gut microbiota assembly (Sloan et al., 2006). Being derived from Hubbell’s neutral theory (Hubbell, 2001), Sloan’s model can recognize the competitive status of a species in a community, and is suitable for assessing a large population size like microbiota community. Observed OTU distributions and mean relative abundances in each of the five populations were fit to this model by the R code^7^ respectively (Burns et al., 2015). To run these scripts, the packages including remotes, Hmisc, devtools, mle, stats4, phyloseq, and DanielSprockett/reltools were installed and loaded. Logistic regressions were performed using the presence/absence of taxa and the partition type to identify taxa above or below the indicated partitions. The average abundance of each OTU across all caterpillar individuals in a population was fit to the neutral model by the parameter of migration rate (m), and the fit of m for each population was assessed with a generalized R-squared. Taxa within the 95% confidence intervals were considered to be well predicted by the neutral model. In order to test the difference in composition between the above- and below-partitions of the neutral model, distance-based redundancy analysis was conducted on Jaccard indices.

## Results

### Intestinal Bacteria Communities in Wild Populations of *Helicoverpa armigera* on Maize

The V4 region of the 16S rRNA gene was sequenced to profile the gut bacterial community composition of 72 individual caterpillars from 5 wild populations. The quality filtered sequences were assigned to 3,176 bacterial OTUs at 97% sequence identity, with 533–2,393 OTUs per sample. The rarefaction curve tended to approach the saturation plateau, indicating adequate sequencing coverage for each sample (Supplementary Figure S1).

**Figure 2:**
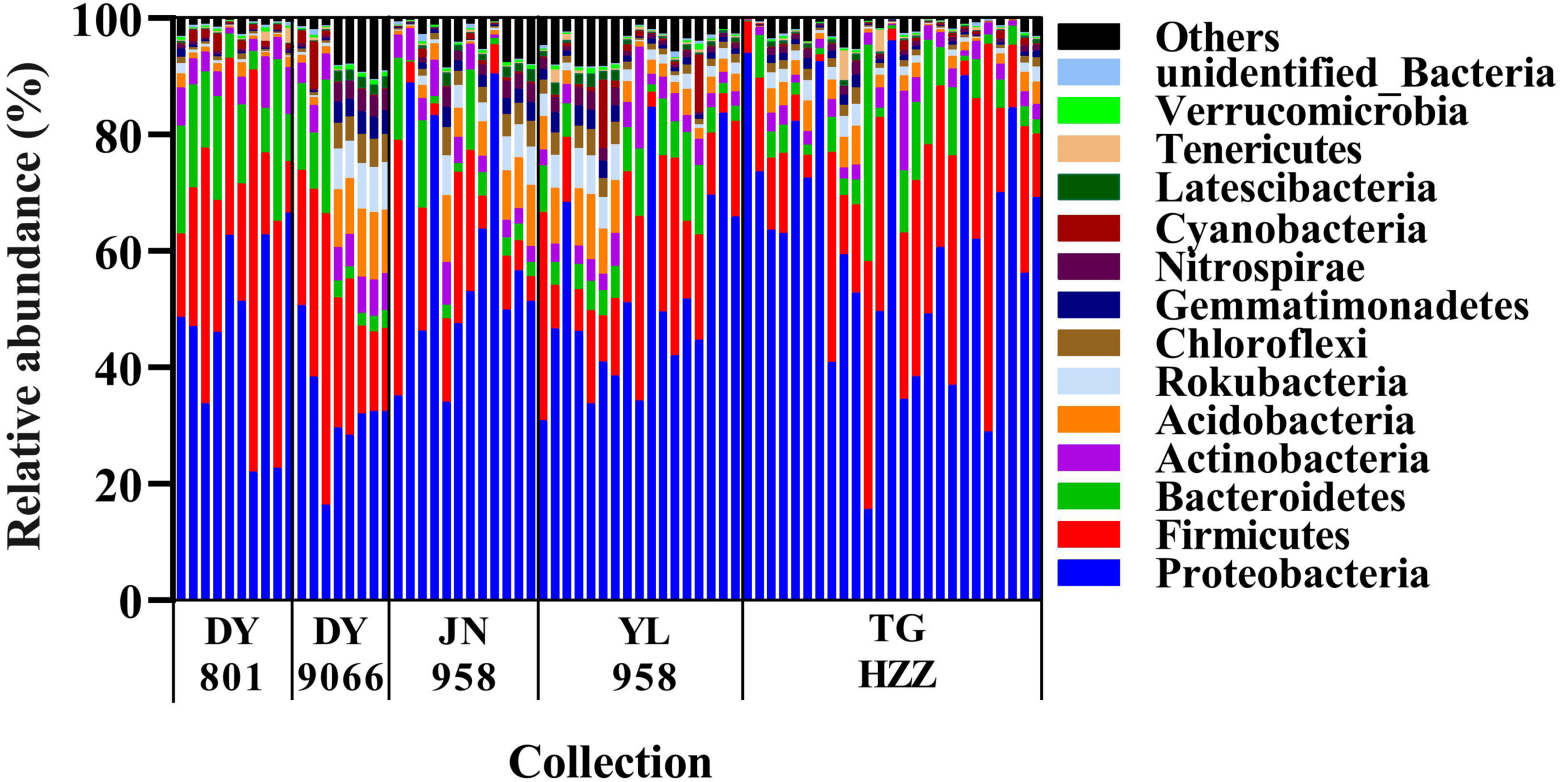
A phylum level perspective on relative abundance of the intestinal bacteria in individual *Helicoverpa armigera* caterpillar. Each column represented an individual caterpillar and the samples were grouped by populations. The top 14 phyla were shown, with the low-abundant phyla being assigned to others.

**Figure 3:**
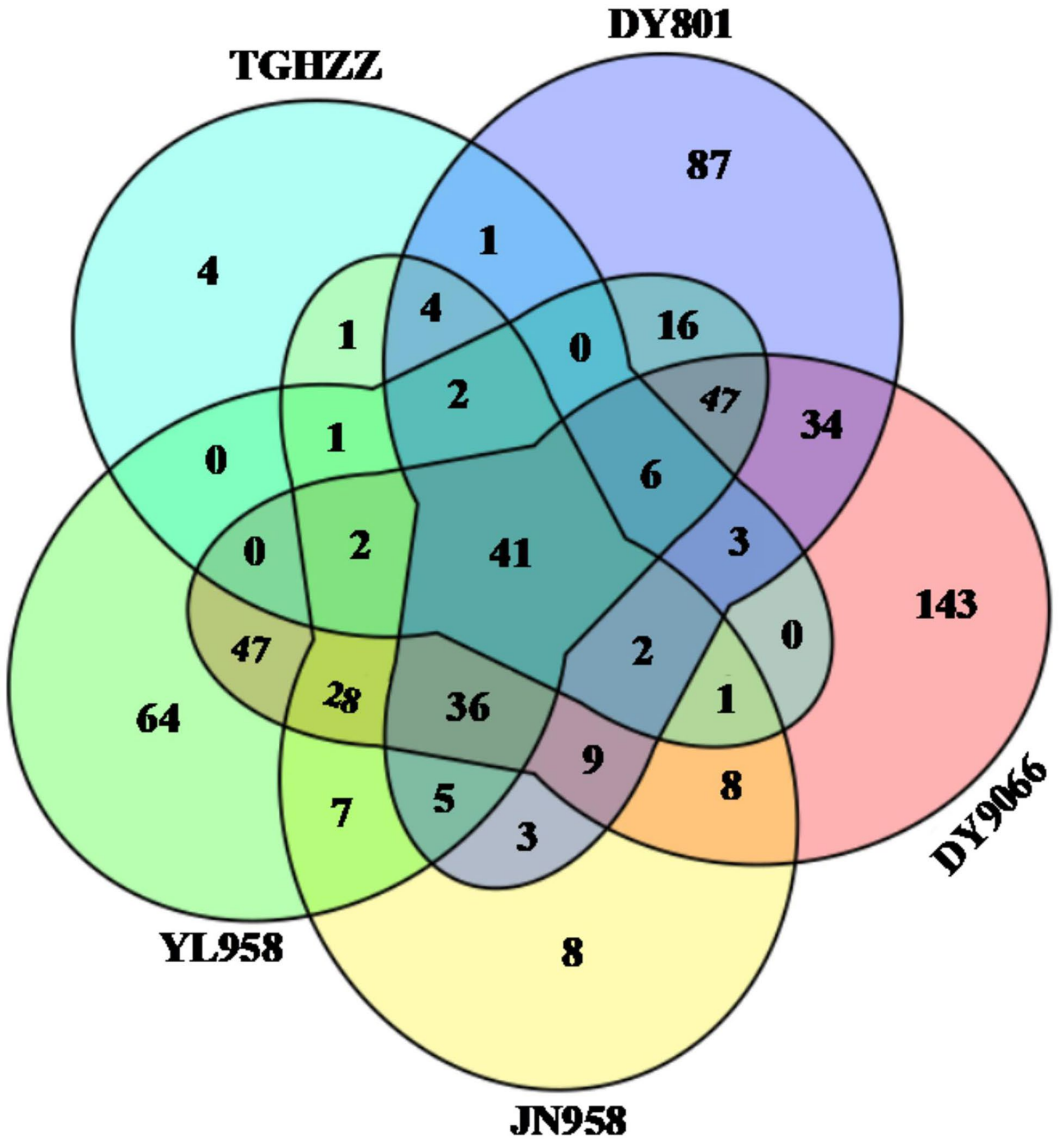
Venn diagram showing the number of unique and shared OTUs in the gut of all *Helicoverpa armigera* caterpillars from the five populations. OTUs shared by all individuals in each of five populations were selected for each population, and then the numbers of unique and shared OTUs among the five populations were presented.

The gut-associated bacteria can be assigned to 37 Phyla, 119 orders, and 515 genera. In total, nearly 90% of the identified bacteria were from five phyla, i.e., Proteobacteria, Firmicutes, Bacteroidetes, Actinobacteria and Acidobacteria (Figure 2; Supplementary Table S2), and the mean relative abundance of each phylum across all samples was 53.44, 20.03, 7.22, 3.85 and 3.65%, respectively. Gammaproteobacteria (31.55%) and Alphaproteobacteria (18.49%) were the two dominant classes in the *H. armigera* caterpillar gut, followed by Bacilli (9.22%), Clostridia (8.76%) and Bacteroidia (7.14%). At the genus level, the top five abundant genera were *Phyllobacterium* (9.80%), *Lactobacillus* (3.55%), *Ralstonia* (3.29%), *Sphingomonas* (3.01%) and *Enterococcus* (2.88%). Moreover, 41 OTUs were shared by all individuals (Figure 3). Nearly half of these OTUs belonged to Proteobacteria, which accounted for 29.05% of total abundance. Other OTUs were from Firmicutes (12 OTUs), Bacteroidetes (3 OTUs), Actinobacteria (2 OTUs), Cyanobacteria (2 OTUs), Rokubacteria (1 OTU), and Acidobacteria (1 OTU; Supplementary Table S3). We randomly selected eight individuals from each population (the number of caterpillars in the smallest collection), and calculated the total number of OTUs and genera in the eight individuals. On average, the microbiota of an individual gut contained 17–75% of the OTUs and 19–79% of the genera in the pooled samples of each population (Supplementary Figure S2).

### Neutral Process Was the Dominant Driver for Microbiome Assembly

**Figure 4:**
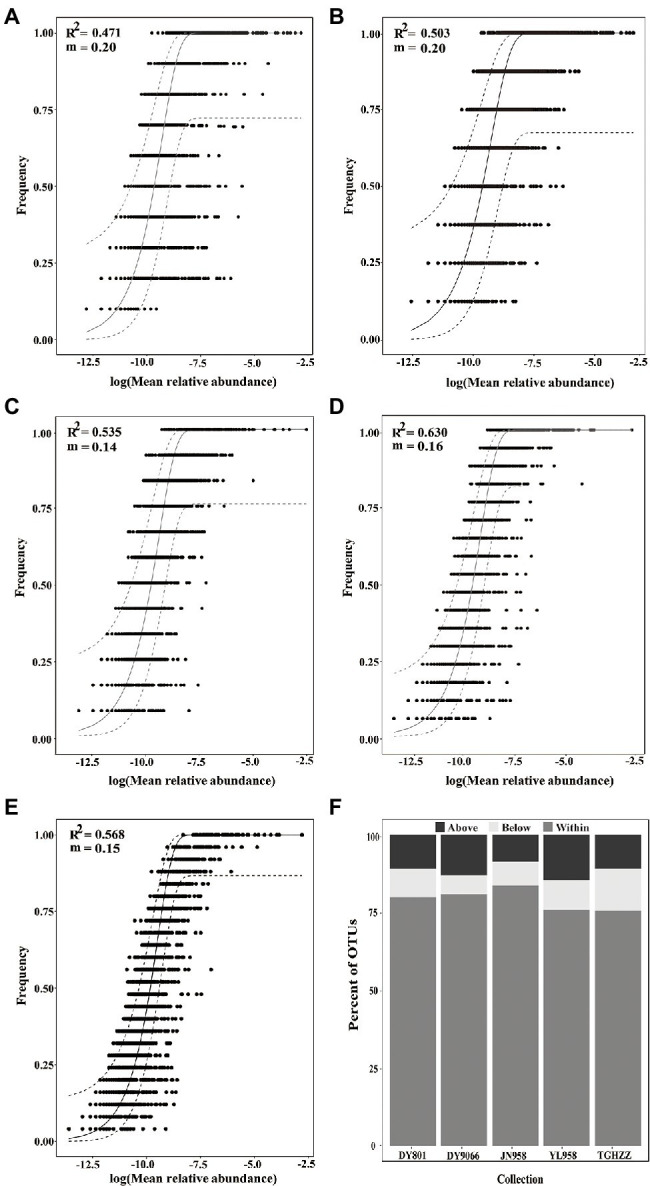
Fit of neutral model for gut-associated bacteria of the *Helicoverpa armigera* caterpillars from 5 wild populations, **(A)** DY801, **(B)** DY9066, **(C)** JN958, **(D)** YL958, **(E)** TGHZZ, and **(F)** the percent of OTUs from each population that fell within, below and above neutral model prediction. An OTU was removed from the analysis if the relative abundance of that OTU in any sample was less than 0.5%. Each point in **(A–E)** represented an OTU in the gut. The solid gray lines represented the best fit to the model and dashed grey lines indicated the 95% confidence interval around the model prediction.

**Figure 5:**
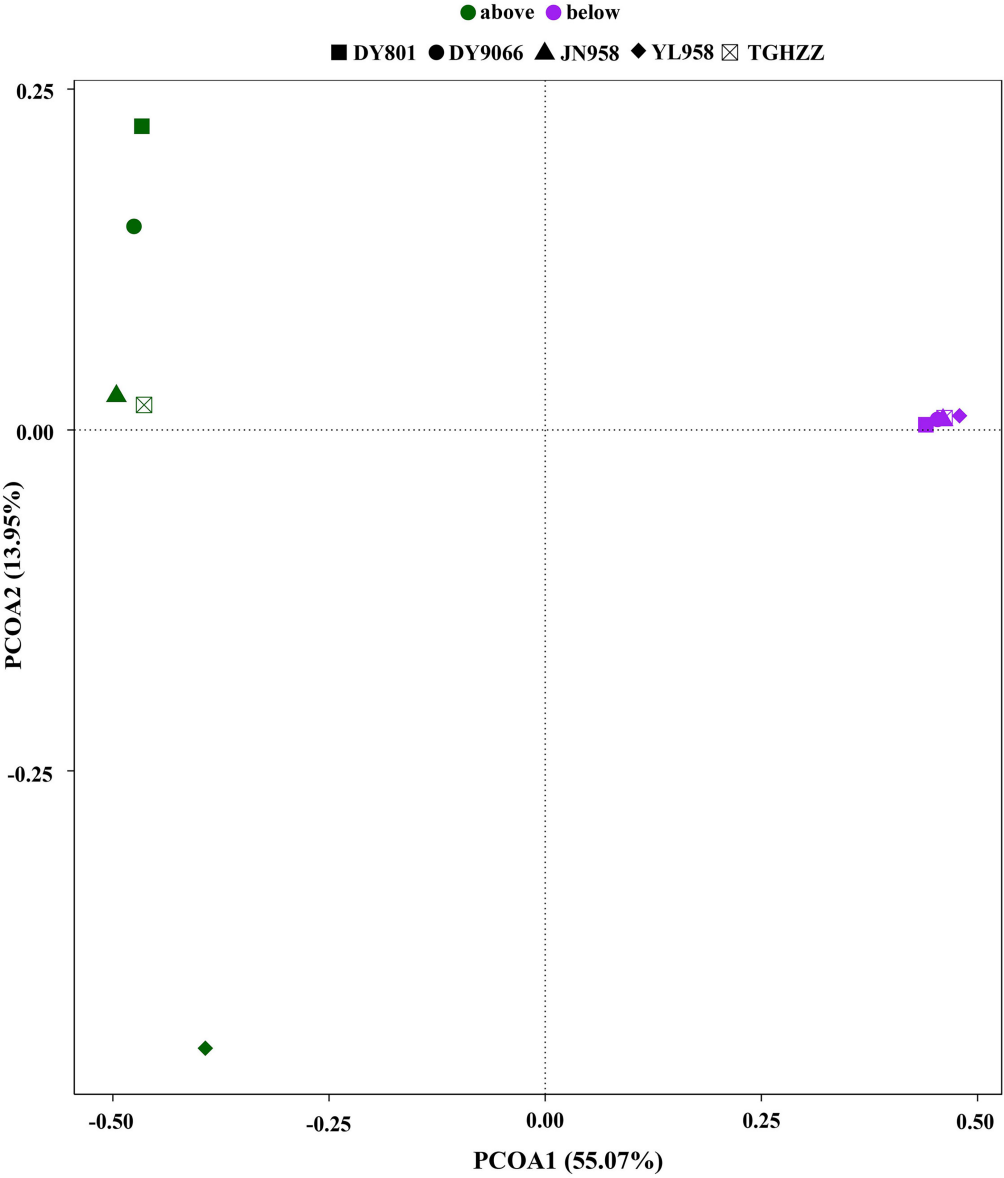
Principal component analysis plot of partitions above and below neutral model predictions based on Jaccard index calculated from the presence/absence of OTUs.

To evaluate the contribution of stochastic processes to the assembly of gut microbiome in the *H. armigera* caterpillars, we fitted the neutral model to the dataset. The neutral model described the frequency distributions of most OTUs (75.59–83.74%) in each population (Figures 4A–E). For the OTUs beyond the 95% confidence limits of each neutral model, 8.56–14.48% were in the above-partitions and 6.04–13.61% were in the below-partitions (Figure 4F). There was a different distribution pattern between the OTUs in the above- and below-partitions. The OTUs in the below-partitions were grouped on a PCoA plot, while the OTUs in the above-partitions were separated (Figure 5). There were 945 OTUs exclusively existing in the within-partitions of the five populations, while no OTU was exclusively found in the above-partitions of all five populations (Supplementary Table S4). Twenty-one OTUs (4 of 5 populations) and 104 OTUs (3 of 5 populations) were found more frequently in the above-partitions than other OTUs among the five populations.

### Both Maize Varieties and Locations Contributed to the Variation of Gut-Associated Bacteria in *Helicoverpa armigera* Wild Populations

To examine whether host plant varieties and geographical locations can contribute to the variation of *H. armigera* microbiota, we conducted two pair-wise comparisons: (1) DY801 and DY9066 populations that were collected from two different maize varieties at a same collection site, and (2) JN958 and YL958 populations that were collected from the single maize variety planted at the two locations far away (Figure 1).

**Figure 6:**
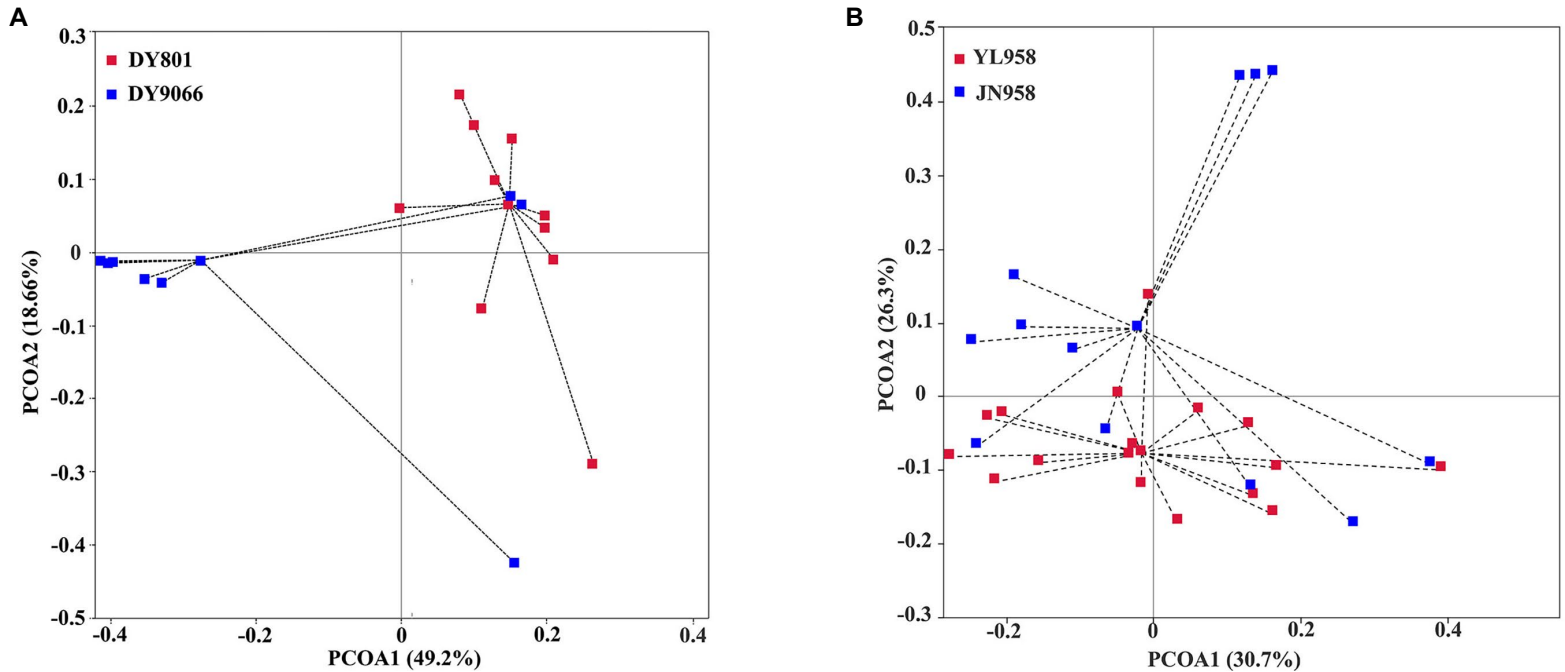
Principal coordinates analyses (PCoA) of the intestinal bacterial microbiota based on weighted Unifrac distances at the OTU level. **(A)** Analysis of two populations on two maize varieties at a same collection site. **(B)** Analysis of two populations on a single maize variety planted at two collection sites. Each point in the Figure represented an individual insect sample, and samples in the same population were indicated by the same color.

**Figure 7:**
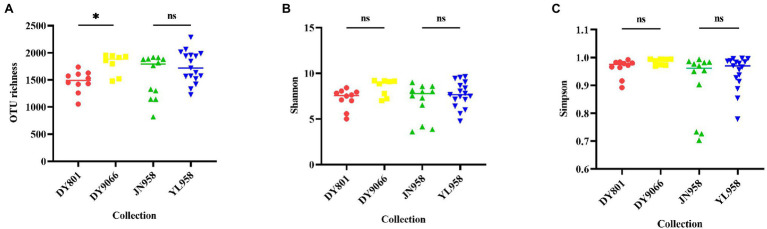
Beeswarm Figures of species richness (OTU number) **(A)**, and community diversity measured by Shannon index **(B)** and Simpson **(C)**. Asterisk above each line represented difference at the significance level of 0.05 (Wilcox rank-sum test) between two populations. ns indicated that there was no significant difference.

**Figure 8:**
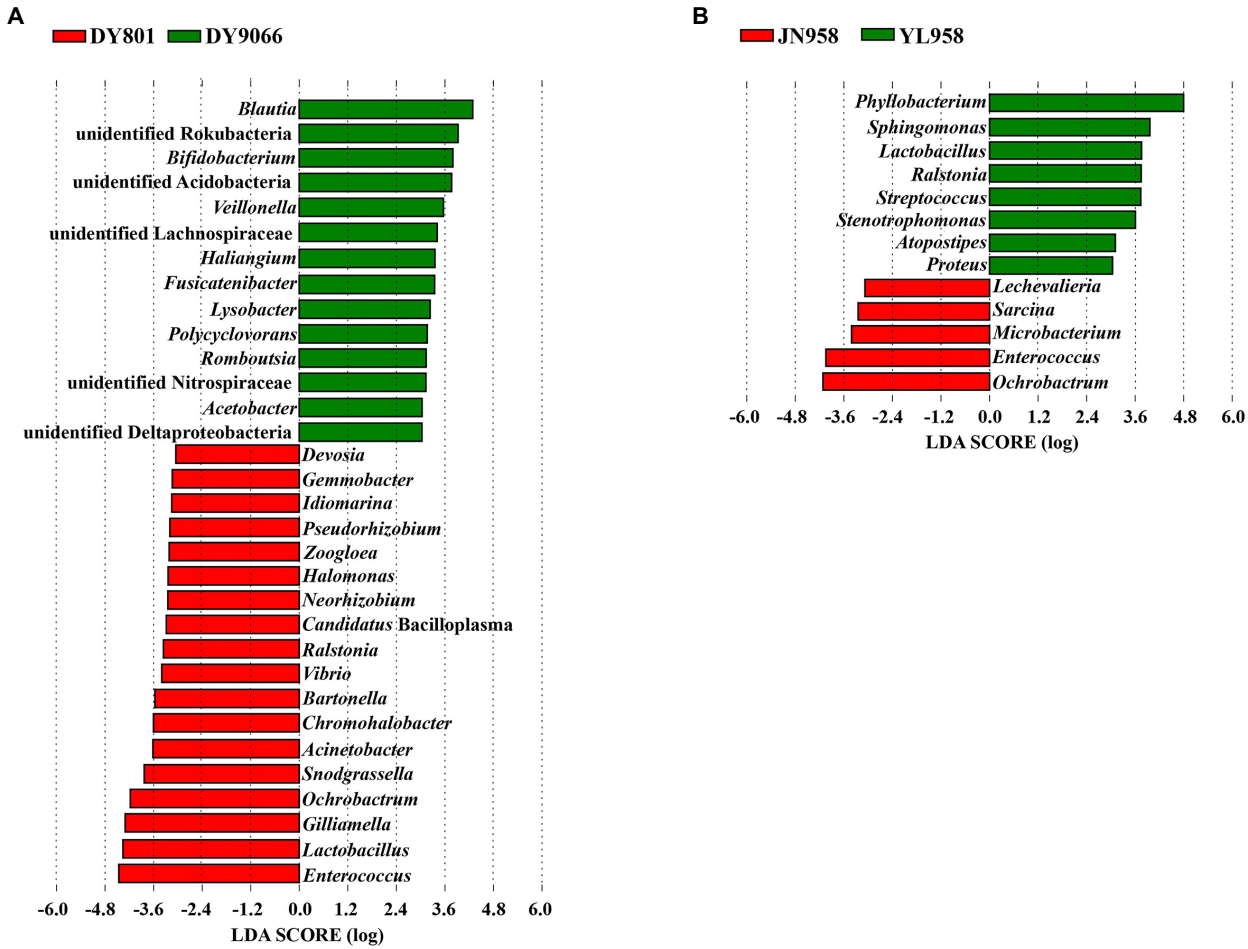
The linear discriminant analysis effect size (LEfSe) analysis of microbial abundance at the genus level. Only the taxa with LDA scores of >3.0 were presented. **(A)** Analysis of two populations on two maize varieties at a same collection site. **(B)** Analysis of two populations on a single maize variety at two collection sites.

Principal coordinate analysis (PCoA) indicated that the gut-associated bacterial communities can be influenced by both maize varieties and geographical locations (Figures 6A,B). Adonis analysis further confirmed that microbiota composition in each pair of comparisons differed significantly (DY801 vs. DY9066: *F* = 4.48, *R*^2^ = 0.219, *p* = 0.004; JN958 vs. YL958: *F* = 2.58, *R*^2^ = 0.087, *p* = 0.008). Moreover, the between-group variation relative to the within-group variation for the pair of DY801 and DY9066 tended to be higher than that for the pair of JN958 and YL958 (Anosim; DY801 vs. DY9066: *r* = 0.49, *p* = 0.002; JN958 vs. YL958: *r* = 0.20, *p* = 0.005). The number of bacterial species differed significantly between DY801 and DY9066, but not between JN958 and YL958 (Figures 7A–C). In total, 32 genera differed significantly in their relative abundance between DY801 and DY9066, with 18 genera and 14 genera being more abundant in DY801 and DY9066, respectively (Figure 8A). In contrast, only 13 genera were significantly different in abundance between JN958 and YL958 (Figure 8B).

## Discussion

As an important agricultural pest, the microbes dwelling inside the gut of *H. armigera* caterpillar were previously characterized by both culture-dependent and culture-independent methods (Mishra and Tandon, 2003; Xiang et al., 2006; Priya et al., 2012; Tang et al., 2012; Ranjith et al., 2016). The top abundant phyla tended to be similar between *H. armigera* populations reared on different plants at different locations. For example, Proteobacteria, Firmicutes, Bacteroidetes, Actinobacteria and Acidobacteria were the major phyla whether the caterpillar was collected from tomato in India or from maize in China. In this study, we evaluated the contribution of neutral processes to the structure of caterpillar gut microbiota, and found that the distribution of a large proportion of gut-associated microbiota in the *H. armigera* caterpillar can be explained by stochastic processes. To our knowledge, the contribution of neutral processes to the structure of insect microbiota has only been investigated in *Drosophila* (Martinson et al., 2017; Adair et al., 2018).

Most gut bacteria fell into the 95% limits of the neutral model, suggesting that a high proportion of the gut-associated bacterial taxa was likely transient. Accordingly, Hammer et al. (2017) found that the surveyed wild caterpillars tended to lack a resident gut microbiome, and most ingested microbes (i.e., microbes in the food) did not colonize in the gut of caterpillars (Hammer et al., 2017). Similarly, the gut microbiome of five *Drosophila* species also fitted well with the predictions of the neutral model (Martinson et al., 2017; Adair et al., 2018). Research on the contribution of neutral assembly processes to microbiota structures in insect hosts is still very limited. Whether bacterial communities of other insects behave according to neutral expectations warrants further investigation. However, there are varying cases reported for non-insect species. For example, in the nematode *Caenorhabditis elegans*, a well-known bacterial feeder, a large number of microbial species deviated from the neutral expectation (both above- and below-partitions; Sieber et al., 2019). These worms naturally feed on bacteria and can likely have strong influence on their microbiota through host defense system. Interestingly, observed and expected patterns in vertebrate microbiota are more complex due, in part, to their more sophisticated immune response systems. Li and Ma (2016) concluded that human microbial communities, covering the five major body sites such as airways, gut, oral, skin, and urogenital, were not neutral in general, by observing that more than 99% of 7,437 datasets from the human microbiome project data center deviated from the neutral expectation (Li and Ma, 2016). In contrast, the intestinal microbiota of a wild *Mus musculus* population collected from 34 unique locations in Bavaria, Germany showed a very good alignment with the neutral expectation (Wang et al., 2015; Sieber et al., 2019). Moreover, the host status can influence the fit of the neutral model. For example, heathy lung microbiota largely followed the pattern of neutral processes but microbes in diseased lungs did not (Venkataraman et al., 2015).

A number of bacterial taxa deviated from the neutral prediction in our study, indicating that they may be more adapted to, or even selected by, the caterpillar gut environment (Sieber et al., 2019). It has been shown that caterpillars can grow well without gut-associated bacteria (Hammer et al., 2017; Phalnikar et al., 2019), but this does not preclude the potential of some gut-associated bacteria to improve host fitness under certain selection pressures (Spiteller et al., 2000; Takatsuka and Kunimi, 2000; Broderick et al., 2006; Pinto-Tomás et al., 2007; Indiragandhi et al., 2008; Anand et al., 2010; Shao et al., 2017; Xia et al., 2017, 2018; Cassone et al., 2020; Chen et al., 2020). Among the bacteria species in the above-partitions, some taxa repeatedly appeared in different caterpillar populations. Interestingly, the functions of certain species belonging to the genera, *Acinetobacter*, *Enterococcus*, *Massilia* and *Streptomyces*, have been investigated in caterpillars in other studies. A study suggested that a gut-associated strain of *Enterococcus* can enhance insecticide resistance of *Plutella xylostella* to chlorpyrifos by regulating the immune system in this insect (Xia et al., 2018). Bacteria species in the genera of *Acinetobacter* and *Massilia* can assist their caterpillar host, *Galleria mellonella*, to degrade the ingested polyethylene and polystyrene (Cassone et al., 2020; Lou et al., 2020; Jiang et al., 2021a). Although polyethylene and polystyrene do not contain the necessary nutrients for insect growth, the food ingested by *H. armigera* caterpillars inevitably contains these environmental pollutants. Some *Streptomyces* strains have been shown to protect hosts such as caterpillars and other insects from pathogens (Chevrette et al., 2019; Kim et al., 2019). However, not all species in a certain genus may perform similar function. Metagenomics studies may help elucidate the potential for different combinations of closely-associated bacteria taxa (the taxa in the above-partitions) to improve host fitness under different environmental challenges.

The midgut of caterpillars is highly alkaline (Dow, 1984; Johnson and Felton, 1996) and can secret antimicrobial peptides (Jiang et al., 2010). Thus, the bacteria species in the below-partitions were possibly more sensitive to this hostile environment and eliminated quickly once entering the gut lumen. Interestingly, the bacterial taxa below the prediction tended to be similar among individual caterpillars collected from different hosts in various locations, indicating that these taxa may be potentially pathogenic agents to the caterpillars. They may be recurrently building relationship with the caterpillar hosts at various environmental conditions but significantly suppressed by the host immune system in the healthy individuals.

Both the maize variety and location contributed to gut microbiota variation in *H. armigera*, so both plant host and location can also contribute to the structure of gut-associated microbiota. The effect of location appeared to be lower than that of diet. In agreement, *H. armigera* larvae collected from the same plant species at different locations tended to share more similar microbiota (Ranjith et al., 2016). Caterpillars generally acquire gut bacteria from their food and environment (Robinson et al., 2010; Tang et al., 2012; Whitaker et al., 2016; Hammer et al., 2017; Chen et al., 2018; Jones et al., 2019) and most of the bacterial species were transient (as discussed above), so variation in the phyllosphere community may also contribute to the difference seen in the gut-associated microbiota between different maize varieties and locations.

In summary, most OTUs in the *H. armigera* gut followed the distribution of the neutral model with a few deviating from the neutral expectation. These findings indicated that the neutral processes should be considered as one important factor when investigating the composition and structure of host-associated microbial communities in caterpillars. In the future, functions of taxa that consistently deviate from the neutral expectation can be investigated by either functional genomic analysis or culturing experiments, which can substantially advance our understanding of biodiversity, community structure and function of caterpillar microbiomes.

## Data Availability Statement

The datasets presented in this study can be found in online repositories. The names of the repository/repositories and accession number(s) can be found at: https://www.ncbi.nlm.nih.gov/, BioProject: PRJNA769459.

## Author Contributions

XJ, SZ, and SL conceived the ideas and designed methodology. SL, RT, HY, ZC, and SS conducted the sample preparation. SL and XJ analyzed the data. XJ, SL, SZ, and T-XL led the writing of the manuscript. All authors contributed critically to the drafts and gave final approval for publication.

## Funding

This study was supported by the National Natural Science Foundation of China (31872299), the National Key R&D Program of China (2016YFD0300705), and the Introduction of Talent Research Start-up Fund of Northwest A&F University (2452017323).

## Conflict of Interest

The authors declare that the research was conducted in the absence of any commercial or financial relationships that could be construed as a potential conflict of interest.

## Publisher’s Note

All claims expressed in this article are solely those of the authors and do not necessarily represent those of their affiliated organizations, or those of the publisher, the editors and the reviewers. Any product that may be evaluated in this article, or claim that may be made by its manufacturer, is not guaranteed or endorsed by the publisher.

## Footnotes

^1^ http://cutadapt.readthedocs.io/en/stable/

^2^ https://www.arb-silva.de/

^3^ http://www.drive5.com/usearch/manual/uchime_algo.html

^4^ https://www.omicshare.com/tools/Home/Soft/venn

^5^ https://www.R-project.org/

^6^ https://rdrr.io/github/DanielSprockett/reltools/src/R/modeling_tools.R
