# Supplementary material for: Neutral Processes Provide an Insight Into the Structure and Function of Gut Microbiota in the Cotton Bollworm
Source: Front Microbiol. 2022 May 3;13:849637. doi: 10.3389/fmicb.2022.849637 (PMC9113526; doi:10.3389/fmicb.2022.849637)
Supplement: Supplementary file 2 [file Data_Sheet_1.docx]

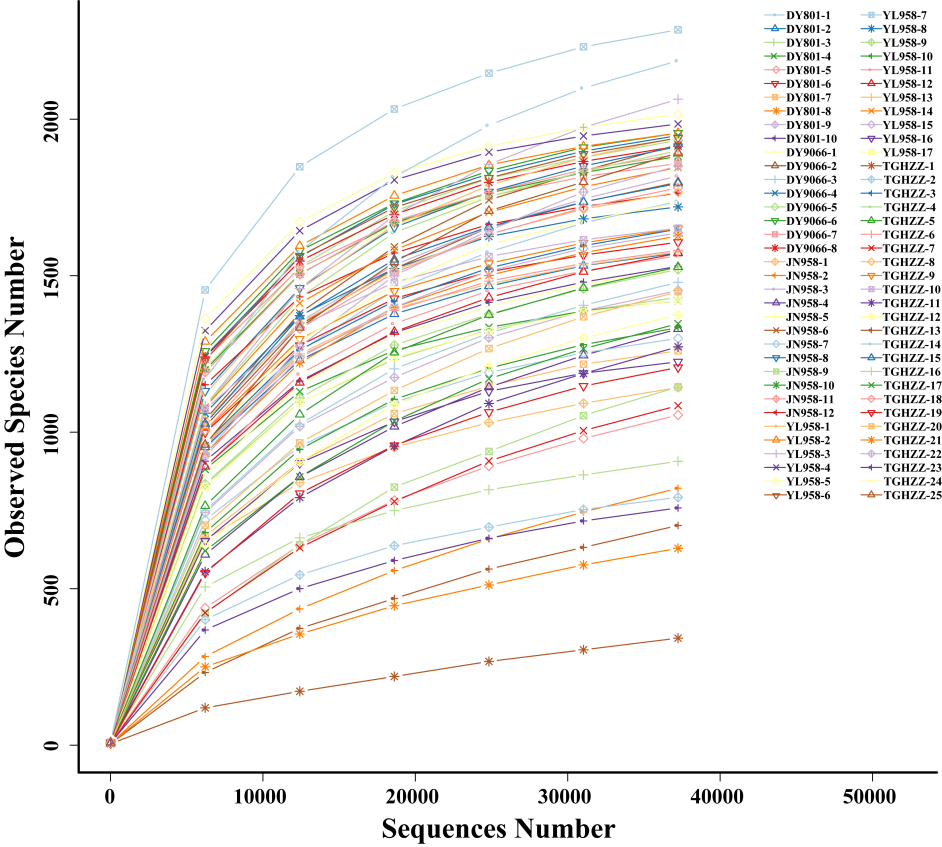


Fig S1. Rarefaction curve of each sample at the 97% similarity level. The X-axis represented the number of sequencing samples randomly drawn from a sample, and the Y-axis represented the number of OTUs that can be constructed based on the number of sequencing sequences to reflect the depth of sequencing. Different samples were shown by curves of different colors.


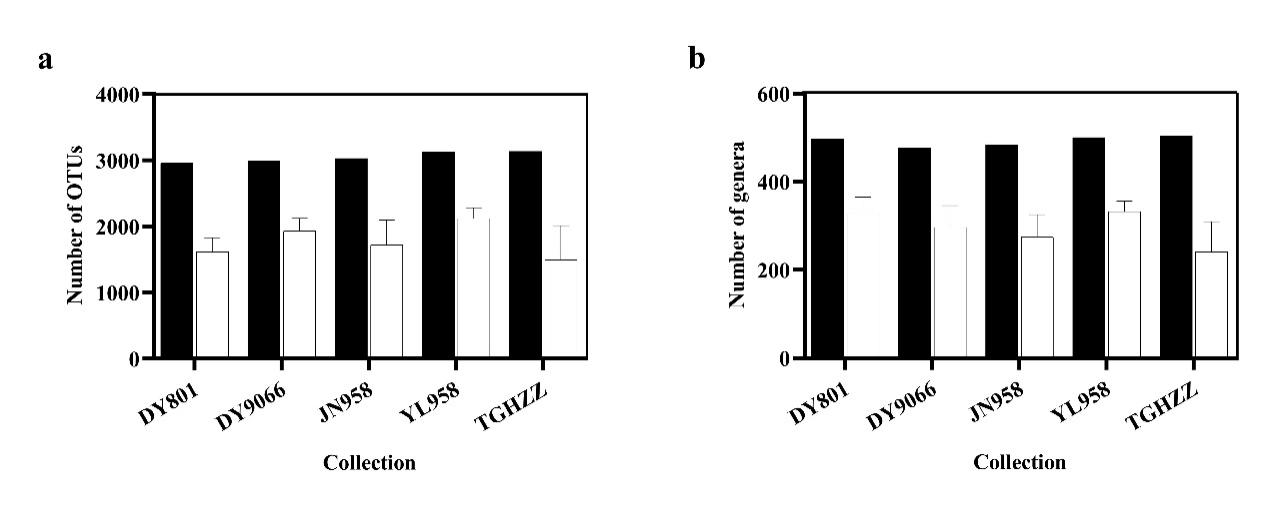


Fig S2. The number of OTUs (a) and genera (b) detected across each population (black bars) and the mean number of taxa (±sd) in the eight randomly chosen individuals (white bars).

Table S1 Sample information

| Sample name | Instar* | Insect mass (mg) | Maize variety | Collection site | Collection time |
| --- | --- | --- | --- | --- | --- |
| DY801-1 | 3 | 58.2 | Ludan 801 | Dong Ying  in Shandong Province | 2018.9.1 |
| DY801-2 | 4 | 184.6 |  |  |  |
| DY801-3 | 4 | 137.1 |  |  |  |
| DY801-4 | 4 | 158.5 |  |  |  |
| DY801-5 | 4 | 187.9 |  |  |  |
| DY801-6 | 3 | 50.2 |  |  |  |
| DY801-7 | 5 | 242.1 |  |  |  |
| DY801-8 | 5 | 217.7 |  |  |  |
| DY801-9 | 3 | 42.3 |  |  |  |
| DY801-10 | 5 | 313.8 |  |  |  |
| DY9066-1 | 3 | 93.4 | Ludan 9066 | Dong Ying  in Shandong Province | 2018.9.1 |
| DY9066-2 | 5 | 173.1 |  |  |  |
| DY9066-3 | 5 | 236.9 |  |  |  |
| DY9066-4 | 3 | 66.5 |  |  |  |
| DY9066-5 | 5 | 333.2 |  |  |  |
| DY9066-6 | 5 | 174.1 |  |  |  |
| DY9066-7 | 4 | 192 |  |  |  |
| DY9066-8 | 3 | 43.2 |  |  |  |
| JN958-1 | 3 | 60.1 | Zhengdan 958 | Ji Nan  in Shandong Province | 2018.9.3 |
| JN958-2 | 4 | 201.1 |  |  |  |
| JN958-3 | 5 | 292 |  |  |  |
| JN958-4 | 5 | 255.4 |  |  |  |
| JN958-5 | 5 | 282.6 |  |  |  |
| JN958-6 | 4 | 144.8 |  |  |  |
| JN958-7 | 5 | 246.2 |  |  |  |
| JN958-8 | 5 | 220 |  |  |  |
| JN958-9 | 5 | 124.1 |  |  |  |
| JN958-10 | 5 | 364.6 |  |  |  |
| JN958-11 | 4 | 194.6 |  |  |  |
| JN958-12 | 5 | 289.2 |  |  |  |
| YL958-1 | 3 | 72.8 | Zhengdan 958 | Yang Ling  in Shaanxi Province | 2018.9.5 |
| YL958-2 | 3 | 83.5 |  |  |  |
| YL958-3 | 3 | 41.1 |  |  |  |
| YL958-4 | 3 | 65.7 |  |  |  |
| YL958-5 | 2 | 29.4 |  |  |  |
| YL958-6 | 4 | 165.6 |  |  |  |
| YL958-7 | 3 | 50.4 |  |  |  |
| YL958-8 | 4 | 154.4 |  |  |  |
| YL958-9 | 3 | 32.4 |  |  |  |
| YL958-10 | 3 | 31.9 |  |  |  |
| YL958-11 | 2 | 66.5 |  |  |  |
| YL958-12 | 5 | 224.8 |  |  |  |
| YL958-13 | 5 | 250.4 |  |  |  |
| YL958-14 | 3 | 574. |  |  |  |
| YL958-15 | 3 | 67.5 |  |  |  |
| YL958-16 | 2 | 24.5 |  |  |  |
| YL958-17 | 2 | 32 |  |  |  |
| TGHZZ-1 | 5 | 511.6 | Heizhenzhu | Tai Gu  in Shanxi Province | 2018.9.13 |
| TGHZZ-2 | 3 | 96.2 |  |  |  |
| TGHZZ-3 | 4 | 153.7 |  |  |  |
| TGHZZ-4 | 5 | 478.5 |  |  |  |
| TGHZZ-5 | 3 | 57.8 |  |  |  |
| TGHZZ-6 | 4 | 208.6 |  |  |  |
| TGHZZ-7 | 5 | 446.5 |  |  |  |
| TGHZZ-8 | 5 | 369.1 |  |  |  |
| TGHZZ-9 | 4 | 233.6 |  |  |  |
| TGHZZ-10 | 4 | 199.3 |  |  |  |
| TGHZZ-11 | 3 | 49.1 |  |  |  |
| TGHZZ-12 | 5 | 371.5 |  |  |  |
| TGHZZ-13 | 5 | 433.4 |  |  |  |
| TGHZZ-14 | 5 | 310.4 |  |  |  |
| TGHZZ-15 | 5 | 386.1 |  |  |  |
| TGHZZ-16 | 5 | 421.7 |  |  |  |
| TGHZZ-17 | 5 | 467.9 |  |  |  |
| TGHZZ-18 | 5 | 420 |  |  |  |
| TGHZZ-19 | 5 | 444.5 |  |  |  |
| TGHZZ-20 | 4 | 295.8 |  |  |  |
| TGHZZ-21 | 5 | 341.9 |  |  |  |
| TGHZZ-22 | 5 | 294.6 |  |  |  |
| TGHZZ-23 | 3 | 92.3 |  |  |  |
| TGHZZ-24 | 5 | 236.1 |  |  |  |
| TGHZZ-25 | 2 | 20.3 |  |  |  |

*Caterpillar instar was estimated by comparing with lab-reared insects.

Table S2 The top 10 abundant phyla, classes, orders, families, genera and species in the gut of *Helicoverpa armigera*. Mean relative abundance (%) with standard deviation (sd) was shown

| No | phylum | class | order | family | genus | species |
| --- | --- | --- | --- | --- | --- | --- |
| 1 | Proteobacteria  (53.44, 20.16) | Gammaproteobacteria  (31.55, 20.99) | Enterobacteriales  (17.07, 23.02) | Enterobacteriaceae  (17.07, 23.02) | *Phyllobacterium*  (9.80, 12.95) | *Ochrobactrum pseudogrignonense*  (1.34, 2.78) |
| 2 | Firmicutes  (20.03, 14.71) | Alphaproteobacteria  (18.49, 13.82) | Rhizobiales  (13.29, 12.75) | Rhizobiaceae  (12.35, 12.86) | *Lactobacillus*  (3.55, 7.70) | *Lactobacillus reuteri*  (1.28, 2.34) |
| 3 | Bacteroidetes  (7.22, 6.93) | Bacilli  (9.22, 11.75) | Clostridiales  (8.76, 8.42) | Burkholderiaceae  (4.67, 6.02) | *Ralstonia*  (3.29, 5.78) | *Escherichia coli*  (1.23, 2.21) |
| 4 | Actinobacteria  (3.85, 2.70) | Clostridia  (8.76, 8.42) | Lactobacillales  (8.03, 11.48) | Ruminococcaceae  (4.13, 5.82) | *Sphingomonas*  (3.01, 3.13) | *Prevotella copri*  (0.60, 1.12) |
| 5 | Acidobacteria  (3.65, 3.53) | Bacteroidia  (7.14, 6.89) | unidentified  Gammaproteobacteria  (7.55, 5.95) | Lactobacillaceae  (3.57, 7.70) | *Enterococcus*  (2.88, 6.73) | *Lactobacillus gasseri* (0.52, 0.69) |
| 6 | Rokubacteria  (2.25, 2.42) | Deltaproteobacteria  (3.24, 2.87) | Bacteroidales  (5.92, 6.71) | Lachnospiraceae  (3.40, 3.49) | *Faecalibacterium*  (2.31, 4.39) | *Cercis gigantea*  (0.49, 1.33) |
| 7 | Chloroflexi  (1.52, 1.50) | unidentified  Actinobacteria  (2.68, 2.52) | Sphingomonadales  (3.35, 3.21) | Sphingomonadaceae  (3.35, 3.21) | *Bacteroides*  (1.75, 3.58) | *Acidobacteria bacterium* RBG 16_ 70_10 (0.28, 0.30) |
| 8 | Gemmatimonadetes  (1.20, 1.20) | Negativicutes  (1.48, 3.82) | Xanthomonadales  (2.32, 3.06) | Enterococcaceae  (3.23, 8.12) | *Stenotrophomonas*(1.73, 2.77) | *Bacteroides fragilis*  (0.26, 0.63) |
| 9 | Nitrospirae  (1.13, 1.11) | Nitrospira  (1.11, 1.10) | Pseudomonadales  (1.59, 2.13) | Xanthomonadaceae  (2.22, 2.88) | *Bifidobacterium*  (1.50, 2.20) | *Roseburia inulinivorans*  (0.22, 0.39) |
| 10 | Cyanobacteria  (0.91, 2.00) | unidentified  Gemmatimonadetes  (1.10, 1.12) | Bifidobacteriales  (1.51, 2.20) | Muribaculaceae  (1.94, 3.23) | *Ochrobactrum*  (1.37, 2.84) | Candidate division NC10 bacterium CSP1-5 (0.20, 0.23) |

Table S3 OTUs shared by all *Helicoverpa armigera* individuals in this study. Mean relative abundance (%) with standard deviation (sd) was shown

| OTU  ID | Relative abundance  (sd) | Phylum | Class | Order | Family | Genus | Species | Confidence  (%) |
| --- | --- | --- | --- | --- | --- | --- | --- | --- |
| OTU_1 | 9.90 (16.49) | Proteobacteria | Gammaproteobacteria | Enterobacteriales | Enterobacteriaceae | - | - | 100 |
| OTU_2 | 7.13 (9.31) | Proteobacteria | Alphaproteobacteria | Rhizobiales | Rhizobiaceae | *Phyllobacterium* | - | 81 |
| OTU_3 | 2.45 (4.66) | Proteobacteria | Betaproteobacteria | Burkholderiales | Burkholderiaceae | *Ralstonia* | - | 95 |
| OTU_4 | 2.23 (2.82) | Proteobacteria | Alphaproteobacteria | Sphingomonadales | Sphingomonadaceae | *Sphingomonas* | - | 100 |
| OTU_5 | 1.98 (4.09) | Firmicutes | Bacilli | Lactobacillales | Enterococcaceae | *Enterococcus* | - | 100 |
| OTU_6 | 1.23 (2.21) | Proteobacteria | Gammaproteobacteria | Enterobacteriales | Enterobacteriaceae | *Escherichia* | *Escherichia coli* | 90 |
| OTU_8 | 0.92 (1.86) | Firmicutes | Clostridia | Clostridiales | Ruminococcaceae | *Faecalibacterium* | - | 100 |
| OTU_9 | 0.77 (1.09) | Proteobacteria | Gammaproteobacteria | Xanthomonadales | Xanthomonadaceae | *Stenotrophomonas* | - | 100 |
| OTU_10 | 0.90 (1.08) | Actinobacteria | Actinobacteria | Bifidobacteriales | Bifidobacteriaceae | *Bifidobacterium* | - | 100 |
| OTU_11 | 0.74 (5.39) | Firmicutes | Bacilli | Lactobacillales | Lactobacillaceae | *Lactobacillus* | - | 100 |
| OTU_12 | 0.68 (1.33) | Firmicutes | Clostridia | Clostridiales | Lachnospiraceae | *Blautia* | - | 100 |
| OTU_13 | 1.28 (2.34) | Firmicutes | Bacilli | Lactobacillales | Lactobacillaceae | *Lactobacillus* | *Lactobacillus reuteri* | 98 |
| OTU_15 | 0.57 (1.08) | Bacteroidetes | Bacteroidia | Bacteroidales | Prevotellaceae | *Prevotella* | *Prevotella copri* | 100 |
| OTU_16 | 0.52 (0.69) | Firmicutes | Bacilli | Lactobacillales | Lactobacillaceae | *Lactobacillus* | *Lactobacillus gasseri* | 99 |
| OTU_17 | 0.56 (0.60) | Proteobacteria | Gammaproteobacteria | -- | - | - | - | 96 |
| OTU_19 | 0.62 (2.11) | Bacteroidetes | Bacteroidia | Bacteroidales | Bacteroidaceae | *Bacteroides* | - | 100 |
| OTU_20 | 0.58 (0.65) | Firmicutes | Bacilli | Lactobacillales | Lactobacillaceae | *Lactobacillus* | - | 100 |
| OTU_24 | 0.22 (0.39) | Firmicutes | Clostridia | Clostridiales | Lachnospiraceae | *Roseburia* | *Roseburia inulinivorans* | 100 |
| OTU_25 | 0.27 (1.20) | Firmicutes | Bacilli | Lactobacillales | Streptococcaceae | *Streptococcus* | - | 97 |
| OTU_30 | 0.30 (0.33) | Rokubacteria | - | - | - | - | - | 100 |
| OTU_31 | 0.49 (1.33) | Cyanobacteria | - | - | - | - | - | 100 |
| OTU_32 | 0.35 (0.65) | Proteobacteria | Gammaproteobacteria | Pseudomonadales | Moraxellaceae | *Acinetobacter* | - | 98 |
| OTU_34 | 0.45 (0.62) | Firmicutes | Clostridia | Clostridiales | Peptostreptococcaceae | *Romboutsia* | - | 100 |
| OTU_168 | 0.14 (0.15) | Acidobacteria | Blastocatellia | Blastocatellales | Pyrinomonadaceae | - | - | 100 |
| OTU_848 | 0.50 (1.46) | Proteobacteria | Gammaproteobacteria | Xanthomonadales | Xanthomonadaceae | *Stenotrophomonas* | - | 100 |
| OTU_926 | 0.25 (0.60) | Bacteroidetes | Bacteroidia | Bacteroidales | Bacteroidaceae | *Bacteroides* | *Bacteroides fragilis* | 100 |
| OTU_1109 | 0.09 (0.17) | Proteobacteria | Gammaproteobacteria | Pseudomonadales | Moraxellaceae | Acinetobacter | - | 90 |
| OTU_2061 | 0.87 (1.50) | Proteobacteria | Alphaproteobacteria | Rhizobiales | Rhizobiaceae | *Phyllobacterium* | - | 100 |
| OTU_2668 | 0.19 (0.27) | Proteobacteria | Alphaproteobacteria | Rhizobiales | Rhizobiaceae | *Phyllobacterium* | - | 86 |
| OTU_3105 | 0.49 (0.91) | Firmicutes | Clostridia | Clostridiales | Ruminococcaceae | *Faecalibacterium* | - | 100 |
| OTU_3404 | 0.51 (1.79) | Proteobacteria | Gammaproteobacteria | Enterobacteriales | Enterobacteriaceae | - | - | 100 |
| OTU_4079 | 0.22 (0.90) | Cyanobacteria | - | - | - | - | - | 100 |
| OTU_6734 | 0.40 (0.66) | Proteobacteria | Gammaproteobacteria | Enterobacteriales | Enterobacteriaceae | - | - | 99 |
| OTU_9362 | 0.79 (6.22) | Proteobacteria | Gammaproteobacteria | Enterobacteriales | Enterobacteriaceae | *Serratia* | - | 93 |
| OTU_9965 | 0.31 (0.55) | Proteobacteria | Alphaproteobacteria | Rhizobiales | Rhizobiaceae | *Phyllobacterium* | - | 96 |
| OTU_10184 | 0.13 (0.17) | Proteobacteria | Alphaproteobacteria | Sphingomonadales | Sphingomonadaceae | *Sphingomonas* | - | 100 |
| OTU_10424 | 0.20 (0.37) | Proteobacteria | Alphaproteobacteria | Rhizobiales | Rhizobiaceae | *Phyllobacterium* | - | 91 |
| OTU_11198 | 0.72 (2.38) | Firmicutes | Bacilli | Lactobacillales | Enterococcaceae | *Enterococcus* | - | 96 |
| OTU_12442 | 0.19 (0.25) | Proteobacteria | Alphaproteobacteria | Rhizobiales | Rhizobiaceae | *Phyllobacterium* | - | 94 |
| OTU_12508 | 0.41 (1.06) | Actinobacteria | Actinobacteria | Bifidobacteriales | Bifidobacteriaceae | *Bifidobacterium* | - | 100 |
| OTU_13128 | 0.25 (0.39) | Proteobacteria | Gammaproteobacteria | Enterobacteriales | Enterobacteriaceae | - | - | 99 |

Table S4 The number of populations (0-5) in which an OTU was predicted to be within the 95% confidence interval around the neutral model prediction, and above or below the confidence limits.
